# Musculoskeletal Diseases: Aetiology, Clinical Implications, Rehabilitation and Treatment

**DOI:** 10.3390/jpm15010035

**Published:** 2025-01-18

**Authors:** Giacomo Farì, Andrea Bernetti

**Affiliations:** 1 Department of Experimental Medicine (Di.Me.S.), University of Salento, 73100 Lecce, Italy; andrea.bernetti@unisalento.it; 2 Infradepartmental University Program of Physical and Rehabilitation Medicine, “V. Fazzi” Hospital, ASL Lecce, 73100 Lecce, Italy

## 1. Introduction

Musculoskeletal diseases (MDs) are a wide range of conditions affecting bones, muscles, joints, and connective tissues and are among the leading causes of disability worldwide. The etiology of these diseases can significantly vary, including genetic predisposition, overuse injuries, autoimmune disorders, and age-related degeneration [1,2]. Osteoarthritis (OA) and rheumatological pathologies, such as rheumatoid arthritis and fibromyalgia, are the most frequent musculoskeletal diseases, each characterized by distinct pathophysiological mechanisms. For instance, OA determines the progressive breakdown of cartilage, leading to pain and motor disability [3], while rheumatoid arthritis results from the autoimmune inflammation of the synovium [4]. The clinical implications of MD are significant, impacting mobility, productivity, and overall quality of life [5].

Rehabilitation and therapy strategies for MD include pharmacological, non-pharmacological, and surgical approaches. Pharmacological treatments often involve analgesics, anti-inflammatory drugs, and disease-modifying agents [6], but alongside these, injection therapies have now also been proven effective [7,8]. Non-pharmacological interventions, such as physiotherapy and structured exercise programs, demonstrated efficacy in improving function and reducing pain [9]. Surgical options, such as joint replacement, are reserved for severe cases where conservative treatments fail [10].

The treatment of MD is undergoing significant innovation with the advent of new technologies. Digital health solutions, such as mobile apps and wearable devices, are increasingly being integrated into rehabilitation and management strategies [11,12]. These technologies enable personalized monitoring, biofeedback, and real-time adjustments to therapeutic exercises, enhancing patient adherence and outcomes. Furthermore, advancements like virtual reality, telemedicine, and digital therapeutics are providing novel, effective approaches for pain management and functional recovery. These innovations are not only improving care but also reducing healthcare costs and promoting accessibility, particularly in managing chronic conditions like osteoarthritis and back pain.

## 2. An Overview of Published Articles

Hakam et al. (contribution 1) investigate non-adherence to physiotherapeutic rehabilitation and explore the cross-cultural adaptation of compliance parameters into the German context. The authors emphasize the importance of understanding cultural differences in adherence to rehabilitation programs, as these factors can impact the effectiveness of treatments. They examined the adaptation process of existing compliance measures for the German population, discussing potential barriers to adherence such as socio-cultural influences. The research highlights the need for tailored rehabilitation strategies to improve patient compliance and outcomes. The findings suggest that addressing cultural nuances is critical in optimizing physiotherapy interventions.

The article by Iaconisi et al. (contribution 2) provides a comprehensive review of the clinical and biochemical implications of hyaluronic acid (HA) in musculoskeletal rehabilitation. It highlights the therapeutic potential of HA in treating joint disorders, particularly in osteoarthritis, due to its anti-inflammatory and tissue-regenerative properties. The review also discusses various HA-based therapies, including intra-articular injections and their impact on pain relief, function, and recovery. Additionally, it examines biochemical mechanisms, such as the modulation of joint lubrication and cell signaling pathways. The authors, in line with the current scientific literature [13,14,15], conclude that HA is a valuable option for musculoskeletal rehabilitation, though further research is necessary to optimize its clinical applications.

Another important aspect widely discussed in this Special Issue concerns musculoskeletal pain, which represents an ever-present challenge for healthcare professionals [16,17].

Ryu et al. (contribution 3) investigate the effectiveness of stellate ganglion block (SGB) in treating post-stroke complex regional pain syndrome (CRPS) using bioelectrical impedance analysis (BIA). This retrospective pilot study explored the potential of BIA as a tool for assessing the physiological effects of SGB on patients’ pain and vascular function. The results showed improvements in pain relief and circulation following SGB treatment, indicating its potential as a therapeutic option for CRPS, which is a frequent problem in post-stroke patients [18]. The study suggests that BIA could be a valuable non-invasive method for monitoring treatment efficacy in CRPS patients. However, further research with larger sample sizes is recommended.

Hernandez-Lucas et al. (contribution 4) conduct a systematic review and meta-analysis to assess the effects of back schools on non-specific back pain, which remains a severe and extremely frequent complaint as it affects 60–85% of individuals during their lifetime [19]. The study examines various back school programs, which typically combine education, exercises, and ergonomic advice to alleviate pain and improve function. The findings show that back schools are effective in reducing pain and improving physical function in patients with non-specific back pain. Additionally, they highlight the importance of tailoring these programs to individual needs for better outcomes. The results support back schools as a useful approach in managing non-specific back pain.

Velickovic and Radunovic (contribution 5) explore the use of repetitive transcranial magnetic stimulation (rTMS) in treating fibromyalgia, focusing on the potential benefits of neuronavigation for targeting new brain regions. The study investigates how rTMS, a non-invasive treatment, can modulate brain activity to alleviate fibromyalgia symptoms. The authors suggest that neuronavigation could improve the precision of rTMS, potentially enhancing its efficacy. Their findings support the idea that targeted rTMS may offer a promising therapeutic approach for fibromyalgia patients. Further research is recommended to refine these techniques and confirm their clinical benefits.

## 3. Conclusions

This collection of articles, all of high quality and great interest, explores the theme of this Special Issue in a multidisciplinary and diverse manner. The varied geographical backgrounds and expertise of the authors have undoubtedly enriched the exploration of a global issue like MD management, from diagnosis to treatment. Experimenting with new methods for evaluating and treating the clinical sequelae of MD, particularly pain and disability, remains an ongoing area of scientific research. Notably, all the published studies highlight the importance of a multidisciplinary approach, which is essential for effective management, integrating medical, physical, and psychological support to address the complex needs of patients [20]. Therefore, we hope that this Special Issue serves as a starting point for further research and publications worldwide.

## List of Contributions

1. Hakam, H.; Lettner, J.; Hofmann, H.; Kersten, S.; Muehlensiepen, F.; Becker, R.; Prill, R. Non-Adherence with Physiotherapeutic Rehabilitation—A Cross-Cultural Adaption of Compliance Parameters into German. *J. Pers. Med.* **2023**, *13*, 1353. https://doi.org/10.3390/jpm13091353.
2. Iaconisi, G.; Gallo, N.; Caforio, L.; Ricci, V.; Fiermonte, G.; Della Tommasa, S.; Bernetti, A.; Dolce, V.; Farì, G.; Capobianco, L. Clinical and Biochemical Implications of Hyaluronic Acid in Musculoskeletal Rehabilitation: A Comprehensive Review. *J. Pers. Med.* **2023**, *13*, 1647. https://doi.org/10.3390/jpm13121647.
3. Ryu, J.; Hwang, I.; Lim, S. Use of Bioelectrical Impedance Analysis to Explore the Effectiveness of Stellate Ganglion Block in Patients with Post-Stroke Complex Regional Pain Syndrome: A Retrospective Pilot Study. *J. Pers. Med.* **2024**, *14*, 258. https://doi.org/10.3390/jpm14030258.
4. Hernandez-Lucas, P.; Leirós-Rodríguez, R.; Lopez-Barreiro, J.; García-Soidán, J. The Effects of Back Schools on Non-Specific Back Pain: A Systematic Review and Meta-Analysis. *J. Pers. Med.* **2024**, *14*, 272. https://doi.org/10.3390/jpm14030272.
5. Velickovic, Z.; Radunovic, G. Repetitive Transcranial Magnetic Stimulation in Fibromyalgia: Exploring the Necessity of Neuronavigation for Targeting New Brain Regions. *J. Pers. Med.* **2024**, *14*, 662. https://doi.org/10.3390/jpm14060662.
